# Supplementary material for: N-Acetylcysteine Reduces the Pro-Oxidant and Inflammatory Responses during Pancreatitis and Pancreas Tumorigenesis
Source: Antioxidants (Basel). 2021 Jul 11;10(7):1107. doi: 10.3390/antiox10071107 (PMC8301003; doi:10.3390/antiox10071107)
Supplement: Supplementary file 1 [file antioxidants-10-01107-s001.zip › antioxidants-1219446-supplementary.pdf]

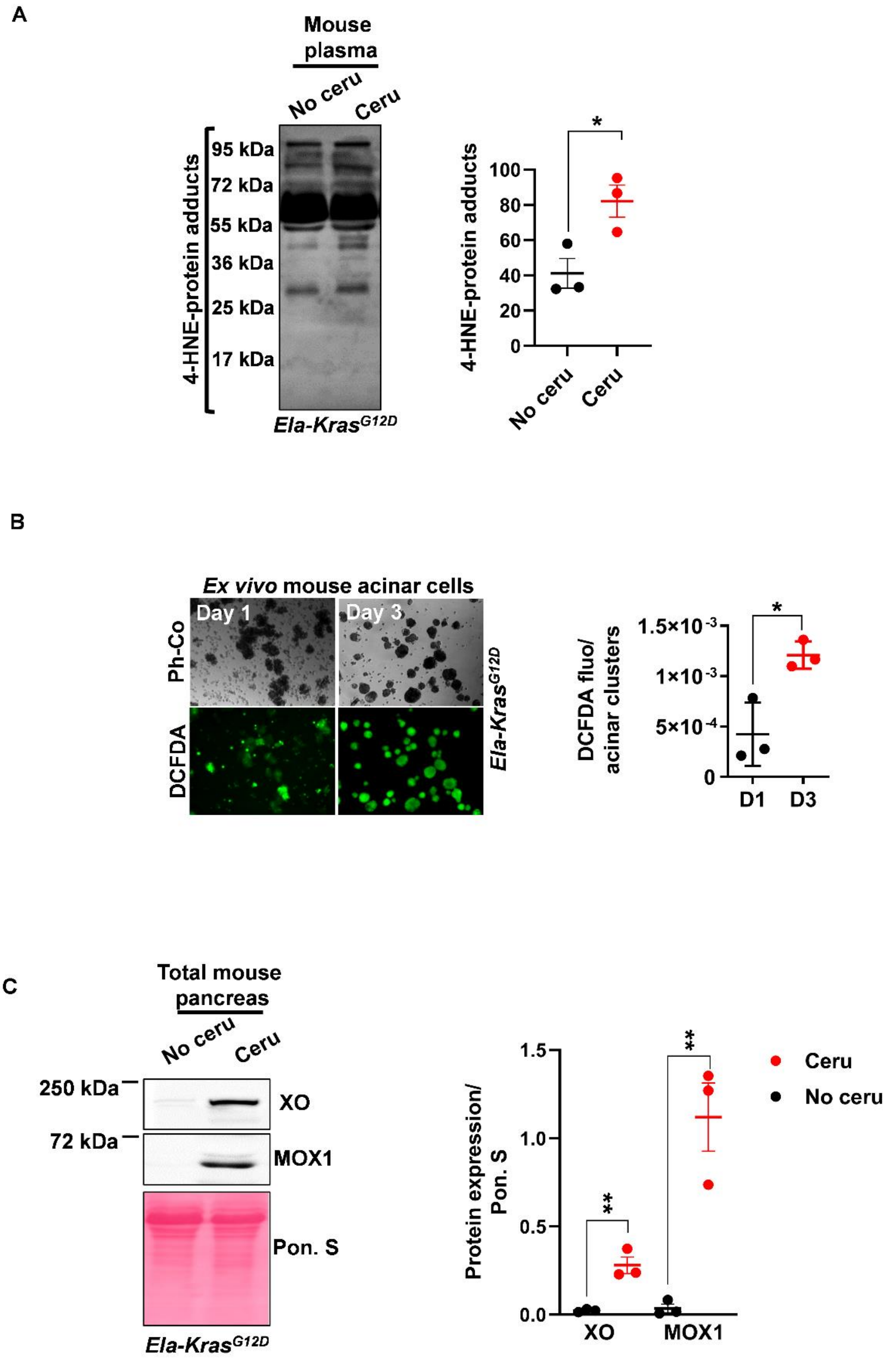

Figure S1. Pancreatitis induces a pro-oxidant response in *Ela-Kras^{G12D}* pancreata. (**A**) Western blot for the oxidative damage marker, 4-hydroxynonenal (4-HNE) on the plasma of *Ela-Kras^{G12D}* mice treated with cerulein for 5 days (n=3) or untreated (n=3). The corresponding densitometry analysis is also available as a bar graph. (**B**) Reactive oxygen species (ROS) were measured in cultured acinar cells *ex vivo* at days 1 (normal) and 3 (metaplasia), using the DCFDA probe; these cells were isolated from *Ela-Kras^{G12D}* pancreata and cultured under conditions mimicking inflammation (n=3). Three independent cultures from three different mice were tested. The quantification of DCFDA fluorescence is available as a bar graph. (**C**) Western blot on pancreas lysates of *Ela-Kras^{G12D}* mice treated with cerulein for 5 days (n=3) or untreated (n=3). The corresponding densitometry analysis is also available as a bar graph. Analyzed enzymes are xanthine oxidase (XO) and mitogenic oxidase-1 (MOX1). Data are mean ± SEM. Statistical significance for all panels was tested by Student t-test (* *P*<0.05, ** *P*<0.01). All quantifications in the figure show biological replicates.

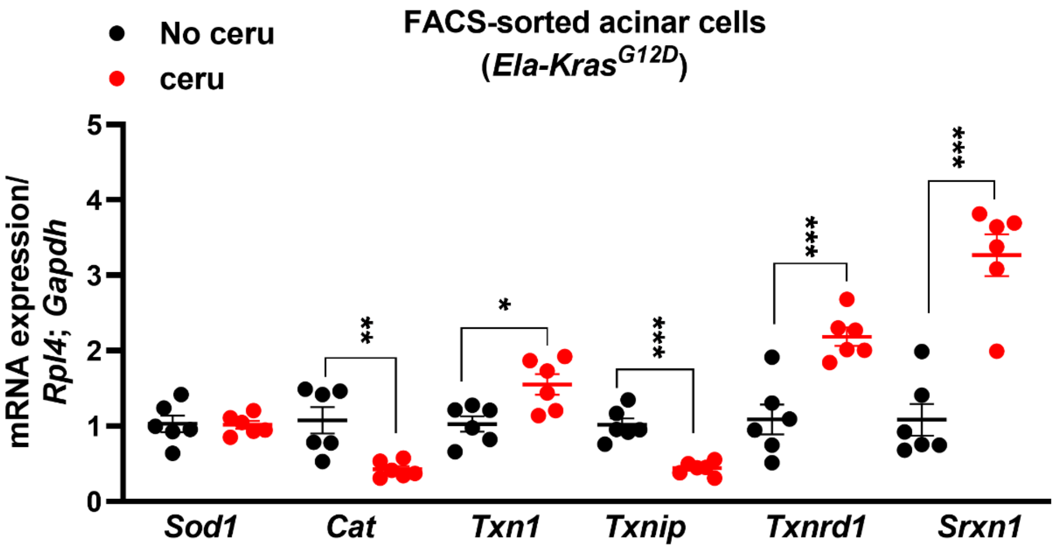

Figure S2. Pancreatitis provokes significant redox changes in pancreatic acinar cells**.** Quantitative PCR analysis for antioxidant genes on FACS-sorted acinar cells from *Ela-Kras^{G12D}* mice treated with cerulein for 3 days (n=6) or untreated (n=6). *Ribosomal protein L4* (*Rpl4*) and *glyceraldehyde-3-phosphate dehydrogenase* (*Gapdh*) were used as housekeeping genes. Data are mean ± SEM. Statistical significance was tested by Student t-test (* *P*<0.05, ** *P*<0.01, *** *P*<0.001). All quantifications in the figure show biological replicates.

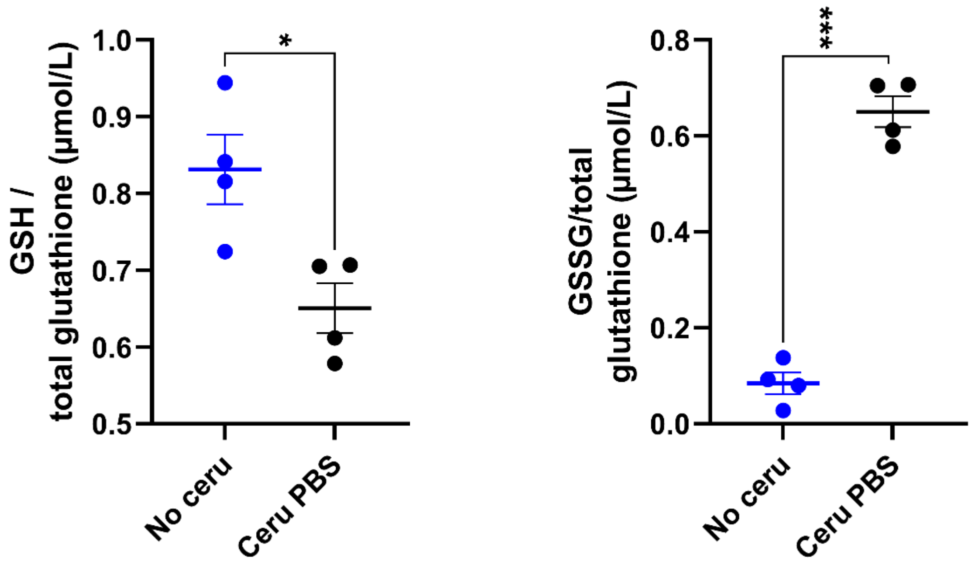

Figure S3. Pancreatitis induces glutathione oxidation in the pancreas. Reduced glutathione (GSH) and disulfide glutathione (GSSG) levels in pancreas lysates of *wild-type* mice treated with cerulein for 1 day (Ceru PBS; n=4) or untreated (No ceru; n=4). Data are mean ± SEM. Statistical significance for all panels was tested by Student t-test (* *P*<0.05, *** *P*<0.001). All quantifications in the figure show biological replicates.

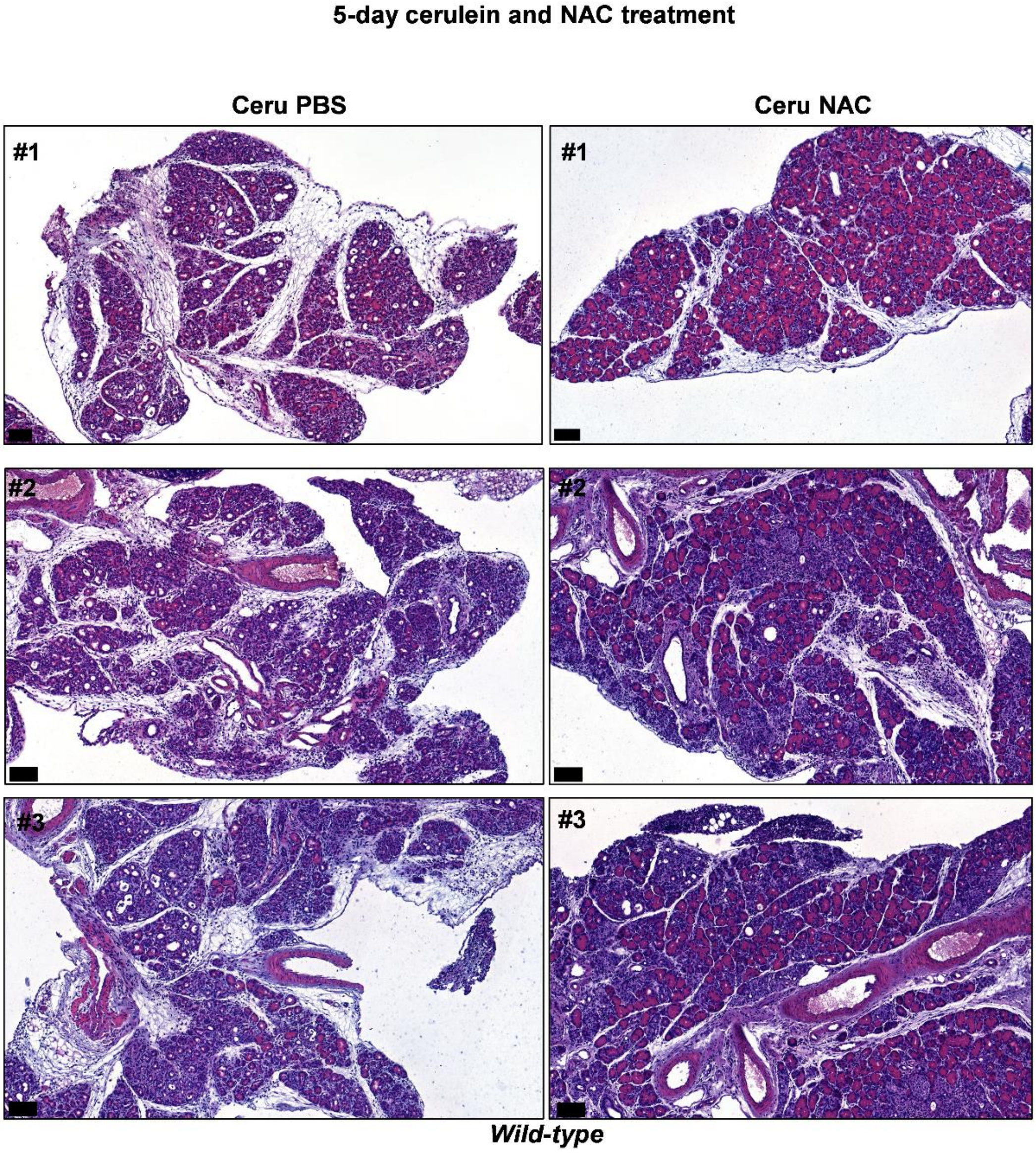

Figure S4. N-acetylcysteine treatment reduces pancreatic edema in *wild-type* pancreata during pancreatitis. Individual tissue scans of hematoxylin and eosin (H&E) staining showing the whole pancreas architecture of *wild-type* mice treated with cerulein and PBS for 5 days (Ceru PBS) or cerulein and NAC for 5 days (Ceru NAC). Three images are displayed for each group. Bars: 200 μm. These scans are related to the edematous area quantification shown in the bar graph of Figure 4D-E. Images are biological replicates.

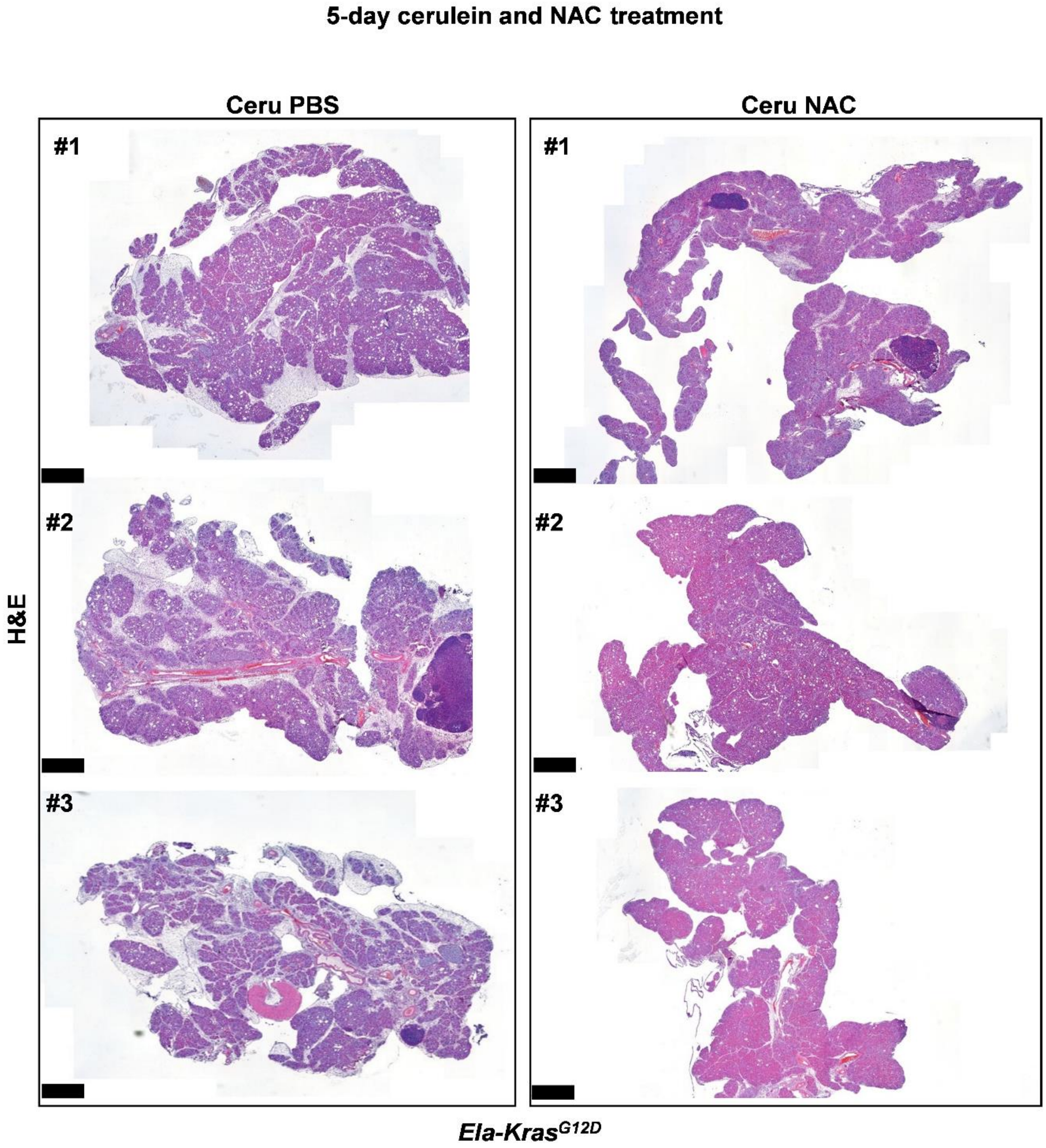

Figure S5. N-acetylcysteine treatment reduces pancreatic edema in *Ela-Kras*^{G12D} pancreata during pancreatitis. Individual tissue scans of hematoxylin and eosin (H&E) staining showing the whole pancreas architecture of *Ela-Kras*^{G12D} mice treated with cerulein and PBS for 5 days (Ceru PBS) or cerulein and NAC for 5 days (Ceru NAC). Three images are displayed for each group. Bars: 1000 μm. These scans are related to the edematous area quantification shown in the bar graph of Figure 5D-E. Images are biological replicates.

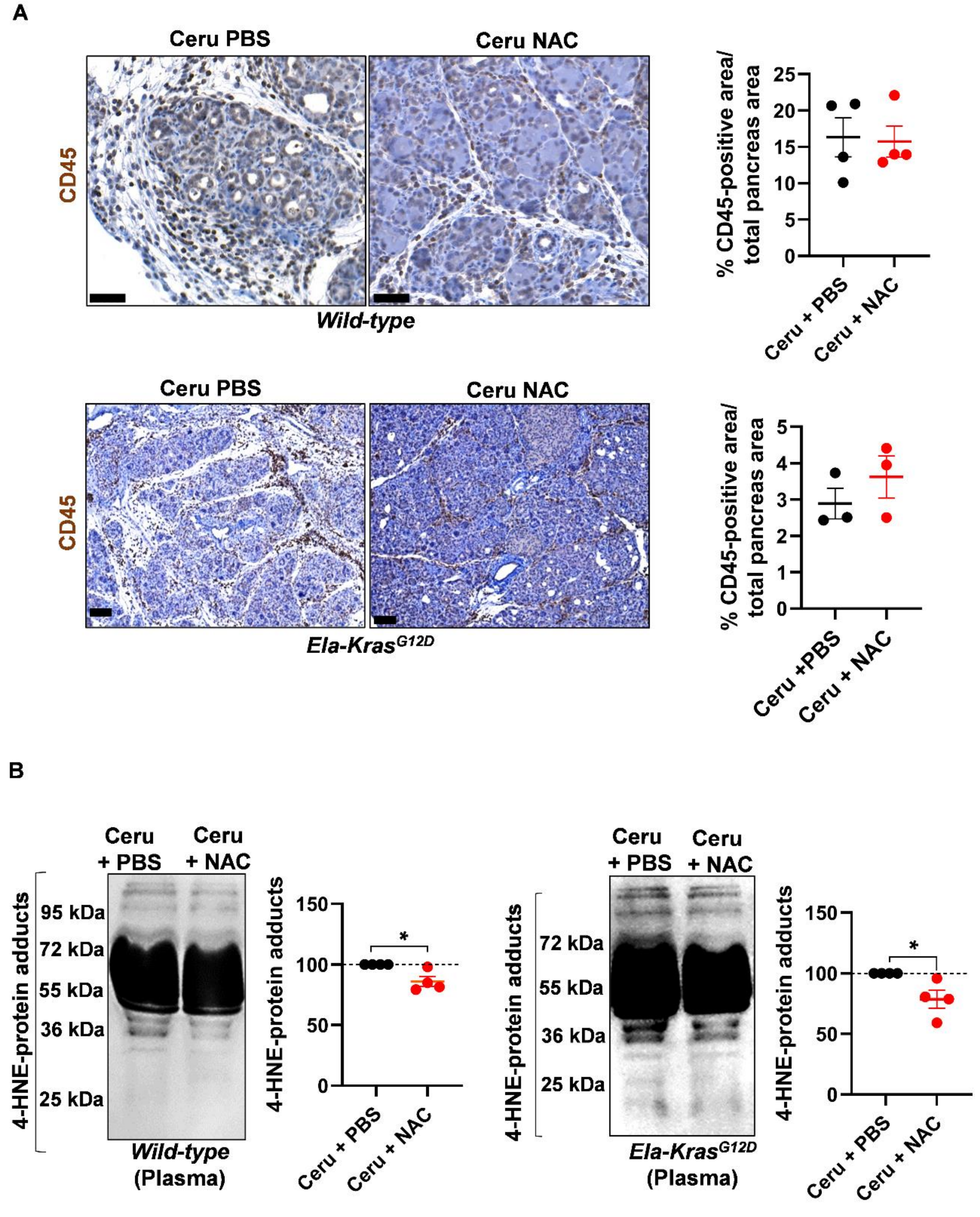

Figure S6. N-acetylcysteine treatment reduces systemic oxidative damage in *wild-type* and *Ela-Kras*^{G12D} pancreata during pancreatitis. (**A**) CD45 immunohistochemistry on the pancreata of *wild-type* and *Ela-Kras*^{G12D} mice treated with cerulein and PBS for 5 days (Ceru PBS; n=4 for both *wild-type* and *Ela-Kras*^{G12D}) or cerulein and NAC for 5 days (Ceru NAC; n=4 for both *wild-type* and *Ela-Kras*^{G12D}). Bars: 50 µm. The corresponding quantification is also displayed as a bar graph. (**B**) Western blot for the oxidative damage marker, 4-hydroxynonenal (4-HNE) on the plasma of *wild-type* and *Ela-Kras*^{G12D} mice treated with cerulein and PBS for 5 days (Ceru PBS; n=4 for both *wild-type* and *Ela-Kras*^{G12D}) or cerulein and NAC for 5 days (Ceru NAC; n=4 for both *wild-type* and *Ela-Kras*^{G12D}). The corresponding densitometry analysis is also available as a bar graph. Data are mean ± SEM. Statistical significance for all panels was tested by Student t-test (* P<0.05). A statistical tendency was considered when P-values were $0.05 > P < 0.1$. All quantifications in the figure show biological replicates.

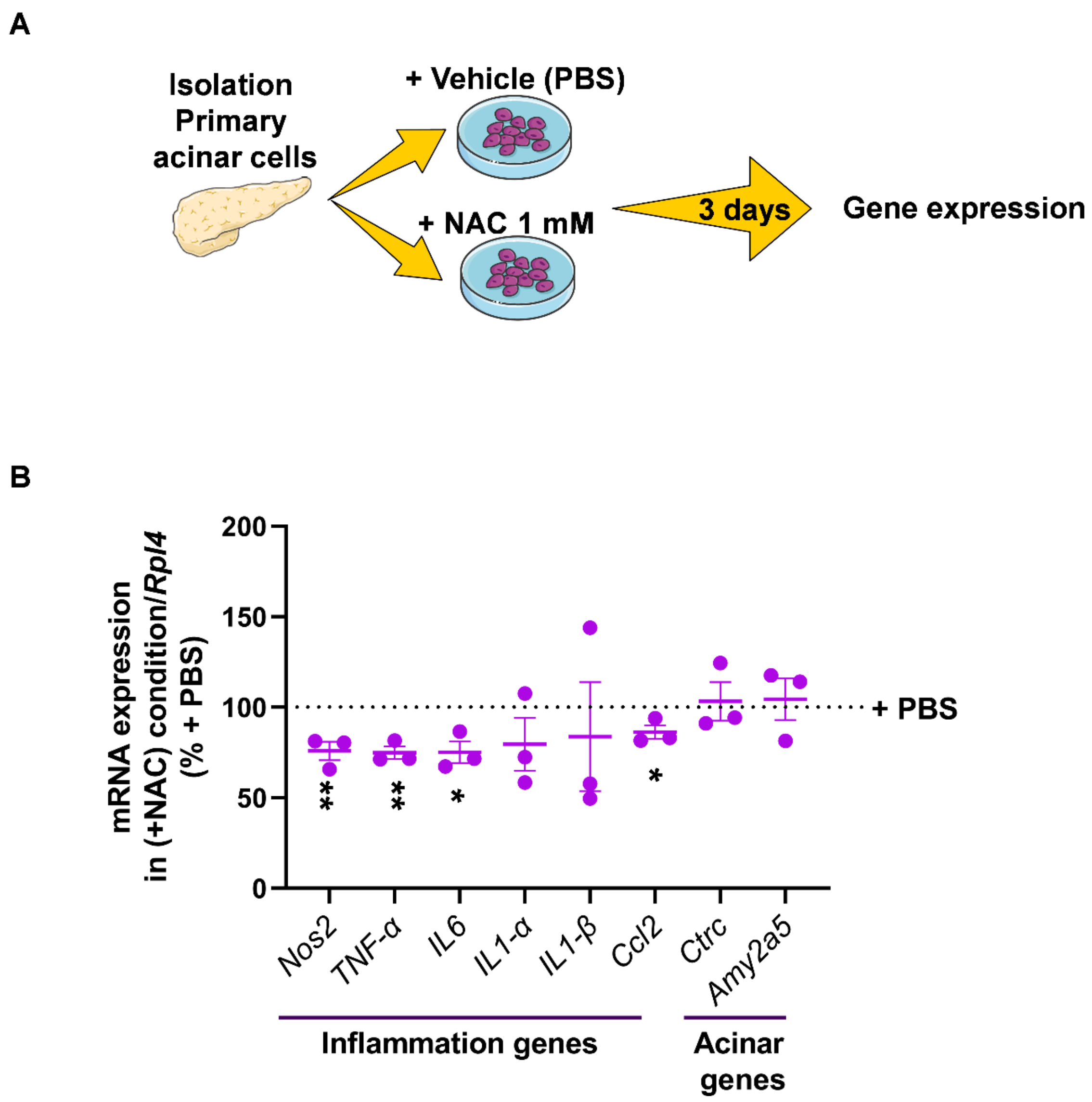

Figure S7. N-acetylcysteine treatment reduces the expression of pro-oxidant and pro-inflammatory genes in primary acinar cells from *Ela-Kras*^{G12D} pancreata. (**A**) Illustration of the used *ex vivo* acinar cell culture. Acinar cells were isolated from the pancreata of *Ela-Kras*^{G12D} mice and then cultured for 3 days (metaplastic cells). Cells were treated with either NAC (1 mM) or vehicle (PBS) from the onset of the culture and until 3 days. (**B**) Quantitative PCR analysis for different pro-oxidant and inflammatory genes on PBS- or NAC-treated acinar cells treated for 3 days (n=3). *Ribosomal protein L4* (*Rpl4*) was used as housekeeping genes. Data are mean ± SEM. Three independent cultures from three different mice were tested. Statistical significance was tested by Student t-test (* P<0.05, ** P<0.01). The dotted line represent the expression level in PBS-treated acinar cells, which was set at 100%. All quantifications in the figure show biological replicates.
